# Programmatic variation in home hemodialysis in Canada: results from a nationwide survey of practice patterns

**DOI:** 10.1186/2054-3581-1-11

**Published:** 2014-06-10

**Authors:** Robert P Pauly, Paul Komenda, Christopher T Chan, Michael Copland, Azim Gangji, David Hirsch, Robert Lindsay, Martin MacKinnon, Jennifer M MacRae, Philip McFarlane, Gihad Nesrallah, Andreas Pierratos, Martin Plaisance, Frances Reintjes, Jean-Philippe Rioux, John Shik, Andrew Steele, Rod Stryker, George Wu, Deborah L Zimmerman

**Affiliations:** Department of Medicine, Division of Nephrology, Northern Alberta Renal Program and the University of Alberta Hospital, University of Alberta, Edmonton, AB Canada; Department of Medicine, Section of Nephrology, University of Manitoba, Winnipeg, MB Canada; University Health Network, University of Toronto, Toronto, ON Canada; Division of Nephrology, University of British Columbia, Vancouver General Hospital, Vancouver, BC Canada; Department of Medicine, Division of Nephrology, McMaster University, Hamilton, ON Canada; Department of Medicine, Division of Nephrology, Dalhousie University, Halifax, NS Canada; Director Home Hemodialysis, London Health Sciences Centre, Western University, London, ON Canada; Division of Nephrology, Horizon Heath, St John Regional Hospital, St John, NB Canada; Department of Medicine and Department of Cardiac Sciences, Division of Nephrology, Southern Alberta Renal Program, University of Calgary, Calgary, AB Canada; Department of Medicine, Division of Nephrology, St Michaels Hospital, Toronto, ON Canada; The Li Ka Shing Knowledge Institute, Keenan Research Centre, St Michael’s Hospital, and Nephrology Program, Humber River Hospital, Toronto, ON Canada; University of Toronto, Humber River Hospital, Toronto, ON Canada; Departement de Medecine, Service de Nephrologie, Centre Hospitalier Universitaire de Sherbrooke, Universite de Sherbrooke, Sherbrooke, QC Canada; Department of Medicine, Division of Nephrology, Hopital du Sacre-Coeur de Montreal, University of Montreal, Montreal, QC Canada; Department of Medicine, Division of Nephrology, Health Science Centre, Memorial University of Newfoundland, St Johns, Newfoundland Canada; Division of Nephrology, Lakeridge Health, Oshawa, ON Canada; Department of Medicine, Division of Nephrology, University of Saskachewan, Saskatoon, Saskatchewan Canada; Division of Nephrology, Trillium Health Partners, Mississauga, ON Canada; Department of Medicine, Division of Nephrology, Ottawa Hospital and the University of Ottawa, Ottawa, ON Canada

**Keywords:** Intensive hemodialysis, Nocturnal, Short daily, Survey, Practice patterns

## Abstract

**Background:**

Over 40% of patients with end stage renal disease in the United States were treated with home hemodialysis (HHD) in the early 1970’s. However, this number declined rapidly over the ensuing decades so that the overwhelming majority of patients were treated in-centre 3 times per week on a 3-4 hour schedule. Poor outcomes for patients treated in this fashion led to a renewed interest in home hemodialysis, with more intensive dialysis schedules including short daily (SDHD) and nocturnal (NHD). The relative infancy of these treatment schedules means that there is a paucity of data on ‘how to do it’.

**Objective:**

We undertook a systematic survey of home hemodialysis programs in Canada to describe current practice patterns.

**Design:**

Development and deployment of a qualitative survey instrument.

**Setting:**

Community and academic HHD programs in Canada.

**Participants:**

Physicians, nurses and technologists.

**Measurements:**

Programmatic approaches to patient selection, delivery of dialysis, human resources available, and follow up.

**Methods:**

We developed the survey instrument in three phases. A focus group of Canadian nephrologists with expertise in NHD or SDHD discussed the scope the study and wrote questions on 11 domains. Three nephrologists familiar with all aspects of HHD delivery reviewed this for content validity, followed by further feedback from the whole group. Multidisciplinary teams at three sites pretested the survey and further suggestions were incorporated. In July 2010 we distributed the survey electronically to all renal programs known to offer HHD according to the Canadian Organ Replacement Registry.

We compiled the survey results using qualitative and quantitative methods, as appropriate.

**Results:**

Of the academic and community programs that were invited to participate, 80% and 63%, respectively, completed the survey. We observed wide variation in programmatic approaches to patient recruitment, human resources, equipment, water, vascular access, patient training, dialysis prescription, home requirements, patient follow up, medications, and the approach to non-adherent patients.

**Limitations:**

Cross-sectional survey, unable to link variation to outcomes. Competition for patients between HHD and home peritoneal dialysis means that case mix for HHD may also vary between centres.

**Conclusions:**

There is wide variation between programs in all domains of HHD delivery in Canada. We plan further study of the extent to which differences in approach are related to outcomes.

**Electronic supplementary material:**

The online version of this article (doi:10.1186/2054-3581-1-11) contains supplementary material, which is available to authorized users.

## Abstract (fr)

**Problématique:**

Au début des années 70, plus de 40% des patients en insuffisance rénale terminale aux États-Unis étaient traités par hémodialyse à domicile (HDD). Cette proportion a décliné rapidement au cours des décennies suivantes, de sorte que le mode de suppléance pour la majorité des patients est maintenant l’hémodialyse 3 fois par semaine à raison de 3 à 4 heures par séance. Les mauvais résultats obtenus avec cette méthode ont renouvelé l’intérêt pour l’HDD, notamment pour les dialyses intensives incluant la dialyse quotidienne courte (DQC) et l’hémodialyse nocturne (HDN). Étant donné leur nouveauté, il y a peu de données sur les façons de faire avec ces modes de suppléance.

**Objectif:**

Afin de décrire les pratiques actuelles, nous avons réalisé un questionnaire systématique auprès des programmes d’HDD au Canada.

**Design:**

Développement et déploiement d’un outil qualitatif.

**Cadre:**

Programmes d’HDD académiques et communautaires au Canada

**Participants:**

Médecins, infirmières et technologues

**Variables mesurées:**

Approches pour la sélection des patients, le mode de suppléance, les ressources humaines disponibles et le mode de suivi pour chaque programme

**Méthodologie:**

Nous avons développé un outil en trois phases. Un groupe de discussion composé de néphrologues canadiens ayant une expertise en DQC ou HDN ont échangé sur le contenu de l’étude et ont rédigé des questions sur 11 domaines. Trois néphrologues familiers avec tous les aspects de l’HDD ont révisé la validité des questions, puis ont demandé un nouvel avis à tout le groupe de discussion. Des équipes multidisciplinaires provenant de trois sites ont ensuite évalué le questionnaire et ont apporté des suggestions. En juillet 2010, le questionnaire a été distribué électroniquement à tous les programmes qui offrent l’HDD d’après le Registre canadien des insuffisances et des transplantations d’organes.

Les résultats ont été compilés au moyen de méthodes qualitatives ou quantitatives, le cas échéant.

**Résultats:**

80% des centres académiques et 63% des centres communautaires invités ont répondu au questionnaire. Nous avons observé des variations importantes entre les programmes quant au recrutement des patients, aux ressources humaines, à l’équipement, à l’eau, aux accès vasculaires, à l’entraînement des patients, à la prescription de dialyse, aux exigences du domicile, au suivi des patients, à la médication et à l’approche face aux patients non-adhérents.

**Limitations:**

Étude transversale, incapacité d’associer les variations aux issues cliniques. La compétition entre l’HDD et la dialyse péritonéale pour le recrutement des patients entraîne peut-être une variabilité entre les centres dans la composition des groupes de patients en HDD.

**Conclusions:**

Il y a de grandes variations entre les programmes dans tous les domaines concernant l’HDD au Canada. Nous planifions d’étudier dans le futur jusqu’à quel point ces différences sont reliées aux issues cliniques.

## Background

Home hemodialysis has its origins in the earliest era of renal replacement therapy. Forty percent of all end-stage renal disease (ESRD) patients in the United States were dialyzing at home in the early 1970s [1]. Over the following decades, home dialysis fell out of favor being largely replaced by conventional thrice-weekly facility-based hemodialysis with 3.5-4 hour treatments in much of the world. Factors such as the unacceptably high mortality associated with ESRD [2], the negative outcomes of the HEMO study [3], and a growing body of literature espousing the benefits of more frequent, intensive hemodialysis prescriptions have spurred resurgent interest in home-based dialysis treatments [4–8].

The two predominant paradigms of home hemodialysis (HHD) are nocturnal hemodialysis (NHD) where patients typically dialyze 3.5-6 nights per week with 6–8 hour sessions, and short daily hemodialysis (SDHD) consisting of 5–6 treatments per week with 1.5-2.5 hour sessions. Both modalities are associated with physiological, quality of life, and survival benefits compared to conventional, facility-based hemodialysis [4, 5, 9–11]. Logistic and economic considerations dictate that most facility-based hemodialysis units cannot easily deliver NHD or SDHD, so that these therapies are ideally performed in the home environment. Many regional dialysis programs have combined their considerable expertise of providing home-based hemodialysis from an earlier era with contemporary NHD or SDHD prescriptions. Anecdotally, this has resulted in variation in practice patterns pertaining to domains such as patient recruitment, training, and follow-up among HHD programs.

We recently reported on patient and technique survival in a multicenter NHD cohort and found a significant center effect, suggesting that variation in program-level clinical care (in addition to differences in case mix) plays an important role in determining adverse outcomes [12]. Indeed, retention to HHD programs in Canada and the United States varies from 64% to 95% at 12 months, further underscoring the need to characterize both patient- and program-specific variables that may explain these differences [12–14]. Importantly, while patient factors predicting poor technique survival or adverse health outcomes are often not-modifiable, program related factors can often be adapted to reflect practices associated with the best outcomes. To date, a granular catalogue of practice patterns for home hemodialysis has not been undertaken so it remains unclear precisely how much variation exists, and which variables might affect outcomes. Here we report on a systematic survey of HHD practice patterns across Canada.

## Methods

A survey instrument was developed between May and July 2010 in three phases. Phase 1 – a focus group of Canadian nephrologists with clinical experience in providing NHD and/or SDHD discussed the scope of practice patterns to be interrogated. Each of the 10 nephrologists was charged with writing questions for one or more domains pertaining to program recruitment, human resources, equipment, water, vascular access, patient training, dialysis prescription, home requirements, patient follow-up, medications, and non-adherent patients. Phase 2 – all questions were compiled and a draft survey was screened by three nephrologists familiar in all aspects of HHD delivery (D.L.Z., P.K., and R.P.P.) for content revision and completeness. A revised draft was circulated to the entire group for feedback resulting in further editing. Phase 3 – a pilot questionnaire was sent to three test centres where it was reviewed by an HHD nephrologist, a nurse manager, the nursing staff responsible for HHD patient care, and the HHD technologist staff. Feedback was incorporated into the survey instrument by the primary investigators (D.L.Z., P.K., and R.P.P.). In addition to the questions pertaining to the aforementioned domains of HHD care, the final questionnaire also incorporated questions regarding ESRD program information. Program details included the total number of ESRD patients treated, the modality distribution of those patients, as well as the specific distribution of HHD modalities (ie. NHD, SDHD, conventional hemodialysis, or a hybrid prescription). The complete questionnaire is available in Additional file 1.

In July 2010 the survey was sent electronically to all academic and community renal programs known to offer HHD according to the Canadian Organ Replacement Registry (CORR). Programs were encouraged to complete the questionnaires jointly between physicians, nursing staff, and technologists. Frequent reminders (maximum of 3) were circulated to encourage completion; data was collated in February 2011. Summary responses by domain are reported here.

## Results and discussion

Of the 15 academic renal programs and 8 community programs surveyed, 12 (80%) and 5 (63%) completed the survey respectively. Within these programs, the mean (±stddev) prevalent use of home dialysis modalities was 24.4 ± 8.4% (range 8.5-42.8%) of the ESRD population (mean 17.5% peritoneal dialysis [PD], 6.9% HHD) (Table 1). For patients receiving HHD, 68.2% were treated with NHD, 9.9% with SDHD (9.9%); the remainder were treated with a hybrid prescription or conventional thrice weekly HD.

**Table 1:** **Renal program census and modality distribution summary**

Program	Program type	Total dialysis population	In-center conventional HD	PD	Total HHD	Total home (HHD + PD)	In-center >3x/wk HD	In-center 3x/wk NHD	Home NHD ^1^	Home SDHD ^2^	Home non-NHD, non-SDHD
	A = academic										
	C = community		(% total)	(% total)	(% total)	(% total)	(% total)	(% total)	(% HHD)	(% HHD)	(% HHD)
1	A	399	69.9%	26.1%	1.8%	27.9%	2.3%	0%	85.7%	14.3%	0%
2	A	656	62.5%	23.0%	14.5%	37.5%	0%	0%	91.6%	8.4%	0%
3	A	958	69.4%	21.7%	6.6%	28.3%	2.3%	0%	73.0%	1.6%	25.4%
4	A	1267	77.3%	19.7%	2.1%	21.8%	0.9%	0%	33.3%	0%	66.7%
5	A	817	74.2%	19.7%	4.3%	24.0%	1.5%	0.4%	65.7%	20.0%	14.3%
6	A	513	77.0%	18.9%	2.1%	21.0%	1.9%	0%	63.6%	18.2%	18.2%
7	A	191	68.1%	18.8%	12.0%	30.8%	1.0%	0%	91.3%	0%	8.7%
8	A	983	77.4%	16.0%	4.8%	20.8%	0.6%	1.2%	57.4%	4.3%	38.3%
9	A	552	65.2%	14.5%	15.7%	30.2%	3.6%	1.3%	76.5%	17.6%	5.9%
10	A	746	75.6%	13.4%	5.5%	18.9%	5.5%	0%	36.6%	17.1%	46.3%
11	A	549	69.9%	11.8%	8.2%	20.0%	10.4%	0%	24.4%	17.8%	57.8%
12	A	187	91.4%	4.8%	3.7%	8.5%	0%	0%	85.7%	0%	14.3%
13	C	385	54.0%	31.4%	11.4%	42.8%	3.1%	0%	56.8%	40.9%	2.3%
14	C	232	74.1%	22.0%	3.0%	25.0%	0.9%	0%	57.2%	0%	42.8%
15	C	493	75.1%	16.8%	3.4%	20.2%	4.7%	0%	94.1%	0%	5.9%
16	C	415	84.3%	9.6%	2.4%	12.0%	3.6%	0%	81.8%	0%	18.2%
17	C	446	70.4%	9.9%	15.2%	25.1%	4.5%	0%	85.3%	7.4%	7.4%
Mean			72.7%	17.5%	6.9%	24.4%	2.3%	0.2%	68.2%	9.8%	21.9%
Median			74.1%	18.8%	4.8%	24.0%	2.3%	0.0%	73.0%	7.4%	14.3%
Standard Deviation			8.4%	6.6%	5.0%	8.4%	2.6%	0.4%	21.6%	11.2%	20.9%

HD, hemodialysis; NHD, nocturnal hemodialysis; SDHD, short-daily hemodialysis; PD, peritoneal dialysis; HHD, home hemodialysis (consisting of all forms of NHD, SDHD and non-NHD/non-SDHD home hemodialysis).

^1^NHD: consisting of any NHD prescription including thrice weekly, every-other-night, or 4–6 times per week.

^2^SDHD: consisting of any SDHD prescription including 1.5-3.5 hour sessions on 5–7 days per week.

### Program recruitment

The majority of programs (76.5%) reported a specific strategy for recruiting patients to HHD, with 82.4% having a designated education session for patients with advanced CKD. This questionnaire did not assess the nature of these sessions (eg one-on-one versus group sessions). Recruitment aids for HHD included videos (58.8% of programs use this aid), posters (64.7%), targeted modality education classes (64.7%), modality education nurses (70.6%) and discussions about patient eligibility at patient care rounds (70.6%) including active recruitment of failing PD patients (82.4%). All programs reported that <20% of acute/urgent dialysis starts initiate HHD, 94.1% of programs report that <20% of patients followed in pre-dialysis CKD clinics initiate HHD. Also all programs reported that almost no one from their in-centre self-care unit transitions to HHD.

There was considerable variability in which professionals contributed to the initial assessment of potential home patients. Most programs (94.1%) reported physician and nurse assessments and a substantial proportion of programs also incorporated other team members’ input (58.8% and 52.9% of programs including social workers and technologists as part of the routine assessment respectively). Patients were not accepted for home HD training if an exit from the program was anticipated in <6 months (~1/3 of programs) or <12 months (~1/3 of programs). However, almost ¼ of programs would accept patients for HHD training regardless of potential early program exits.

### Human resources

**Figure 1:**
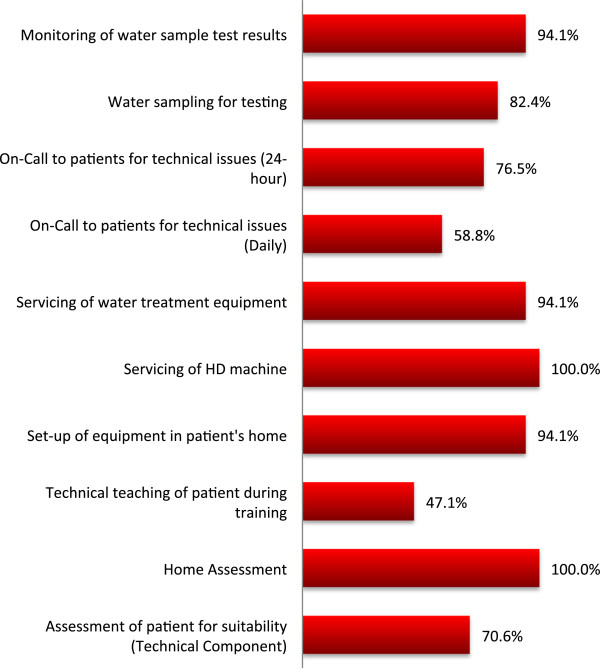
**Duties performed by technologists in HHD.**

The majority of programs (57.9%) reported that some allied health team members worked exclusively in the home dialysis unit (nurses) while others (pharmacists, dieticians, technologists) were cross-appointed to other parts of the global dialysis program. Only 5.3% of programs stated that all allied health team members worked strictly in the HHD unit. The average patient to professional ratio for each of the following health professionals: dieticians (152:1), pharmacists (142:1), social workers (128:1), administrative clerks (82:1), technologists (23:1) and nurses (16:1) for programs with these professionals (eg. not all programs may have had pharmacists). Thirty percent of programs completely outsourced the technologist support required for HHD to a vendor or other source. The majority of programs used a primary nursing model (58.8%) with a shared nephrologist model (84.2%). For the programs with in-house technologists, a wide variety of duties were required of them in different programs (Figure 1).

### Equipment

Conventional HD machines were used by all of the programs with maintenance primarily done by in-house technologists (64.7%). The majority of programs (76.7%) used reverse osmosis equipment; a few used de-ionizers (23.5%). The majority of programs (58.8%) used a post-treatment ultrafilter with some programs adding ultraviolet light (23.5%). The majority of programs (58.8%) provided weigh scales and centrifuges (82.4%).

### Water

**Figure 2:**
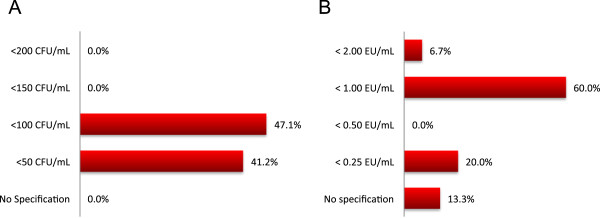
**Programmatic tolerance limits for water quality. (A)** visible microbial counts, and **(B)** maximum endotoxin concentration in product water in patients’ home HD.

The majority of programs (94.1%) have had experience with patients using well water with a minority of programs (35.3%) having had patients using surface water such as ponds or lakes. There was variability in programmatic allowances for visible microbial counts and maximum endotoxin concentration of product water for patients’ homes (Figure 2).

### Vascular access

**Figure 3:**
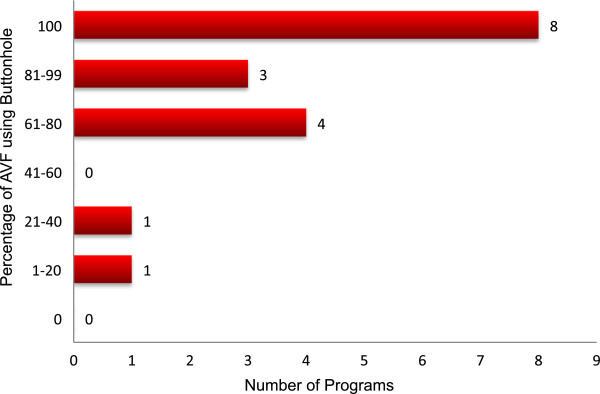
**Programmatic use of buttonhole cannulation.** This graph depicts the number of programs having specific proportions of their AVF patients who use buttonhole cannulation (ie. 8 programs have all of their AVF patients using buttonholes, while 1 program has <20% of its AVF patients using buttonholes).

**Figure 4:**
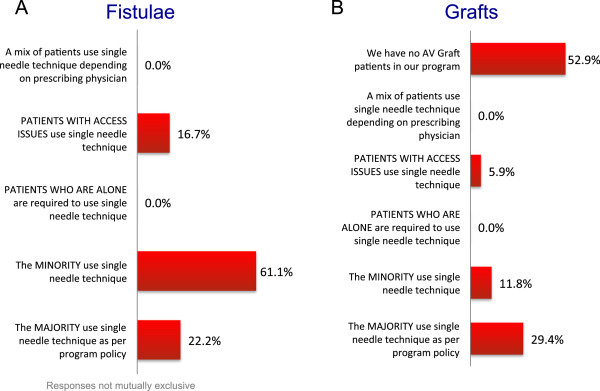
**Usage of single-needle dialysis for patients undergoing NHD among: (A) fistula and (B) graft users.**

The arteriovenous fistula (AVF) was the preferred access for 88.2% of the programs; 11.8% of programs did not identify any type of access as preferable over another. The absence of an AVF did not preclude HHD in any program though it may delay initiation of HHD training in 37.5% of programs. In 8 of 17 programs, buttonhole cannulation was used for all patients with an AVF and all programs used buttonhole cannulation for at least some patients (Figure 3). The majority of programs (64.7%) routinely created 1 set of buttonhole sites (ie. 2 buttonholes) for patients using this cannulation technique; 23.5% created 2 sets. Steel needles were used by >80% of programs irrespective of cannulation method (rope-ladder or buttonhole), while the remainder used Teflon, Supercath or Angiocath needles. Special safety engineered needles were mandated by only 31.1% of programs. There was considerable variability in routine access monitoring: 5.9% of programs never monitor flows, 29.4% monitor every 2–3 months, 23.5% monitor at intervals greater than 3 months, and 52.9% monitor only if clinically indicated (responses are not mutually exclusive). Figure 4 summarizes the usage of single needle dialysis relative to vascular access type (fistula versus graft) for patients specifically receiving NHD; single needle dialysis was relatively more common among patients with grafts compared to fistulae. For patients with a central venous catheter (CVCs), 64.5% of programs used some type of safety connector (eg. TEGO) to prevent air embolism and 35.3% of programs used connection safety devices such as lock boxes; the remainder did not mandate the use of any special safety connectology. CVC’s were locked with citrate or heparin in 64.7% and 35.3% of programs respectively.

### Patient training

**Figure 5:**
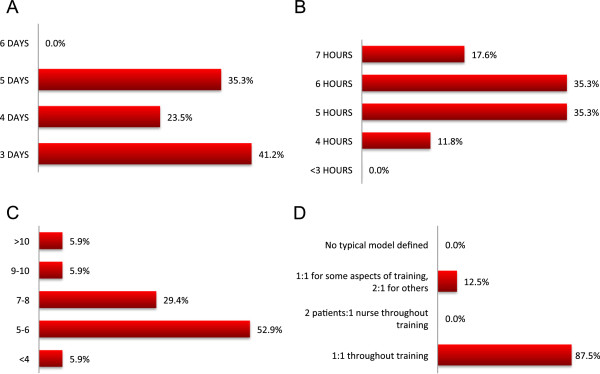
**Training schedule. (A)** Typical number of days per week a program trains patients, **(B)** typical number of hours per days a program trains patients, **(C)** median number of weeks a patient trains for HHD, and **(D)** characteristics of patient-to-nurse ratio during HHD training.

The majority of programs (94.1%) used their own program-generated training manual and 17.6% supplement with a self-created video. Most programs (52.9%) also used industry-generated material. Almost all programs provided 1:1 nurse-to-patient training (87.5%) that occurred 3 (41.2%), 4 (23.5%) or 5 days (35.3%) per week for 4 to 7 hours per day in the HHD unit (Figure 5). The median number of weeks to train a patient varied widely from <4 weeks to >10 weeks. Patients initially educated to use a CVC for HHD required additional training to transition to AVF use at home. There was considerable variability in the amount of time required for access retraining with 1–3 days, 4–6 days, 7–9 days, >10 days, or no specific additional training days allocated by 5.9%, 17,6%, 29.4%, 29.4%, and 11.8% of programs respectively.

All programs required that a nurse and/or technologist accompany a patient during their first self-administered treatment at home; no program allowed patients to treat themselves un-supervised for their first home treatment.

### Dialysis prescription

The starting prescription for NHD included sodium concentration of 136-140 mmol/L, potassium of 2-3 mmol/L, bicarbonate of 28-37 mmol/L, calcium of 1.25-1.75 mmol/L and glucose of 5.55-11.1 mmol/L. The dialysate flow rate ranged from 150-500mls/min with both high and low flux dialyzers being used. The SDHD prescriptions had similar variability in dialysate composition but uses higher dialysate flow rates (500-800 mls/min) and all programs used high flux dialyzers. Few programs prescribed ultrafiltration profiling (17.6% prescribe occasionally, 82.4% never) or blood volume monitoring (11.8% occasionally, 88.2% never).

### Home treatment requirements and costs

**Figure 6:**
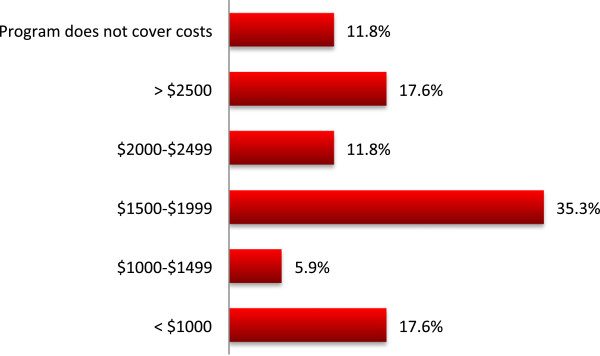
**Estimated average per patient cost for home assessment and home renovations born by the HHD program.**

A home assessment to determine the technical feasibility of conducting HHD was integral to all programs. This was typically conducted by the technologist from the HHD program (41.2%), completely outsourced to a third party such as a dialysis machine vendor (29.4%), or by a combination of technologist and vendor (29.4%). A dedicated nursing assessment of the home was conducted by 58.8% of programs. The majority of programs at least partially reimbursed expenses for minor plumbing and electrical renovations (88.2%) though there is considerable variability in the dollar value (Figure 6). The majority of programs (58.8%) covered this cost only once. The actual renovations are typically outsourced (88.2%) or performed by a combination of program technologist and an outsourced tradesperson (11.8%). Some programs (29.4%) extend their service to a recreational vehicle and/or cottage. The additional expense of UV lights, iron filters, and water softeners is born by 35.3%, 41.2%, and 58.8% of programs respectively; 29.4% of programs report not needing additional water treatment equipment. The additional utility expense incurred by patients dialyzing at home is offset by only 29.4% of programs.

Besides the technical prerequisites to performing HHD, 58.8% of programs required that selected HHD patients had a care partner at home during the treatment, while 41.2% never required such assistants. The majority of care partners were family members or living companions (64.7%) though some programs had experience with paid assistants (47.0%) and unpaid volunteers (11.8%). Remote real-time monitoring of hemodialysis treatments was not routine in any program, with 82.4% of programs reporting never having used this technology and 16.7% of programs utilizing such monitoring only in very specific circumstances.

### Patient follow-up

Although almost all programs (94.1%) had a nurse perform a home visit during the first 1–2 treatments, 41.2% of programs did not schedule subsequent home visits except on an as-needed basis with only 35.3% having a fixed home visit schedule; the remaining programs did not perform follow-up nursing home visits. An audit of patient technique was typically done only if there is an adverse event at home (41.2%) or at the specific request of the nurse/nephrologist (35.3%); annual patient “recertification” was routine in only 2 of 17 (11.8%) of programs.

**Figure 7:**
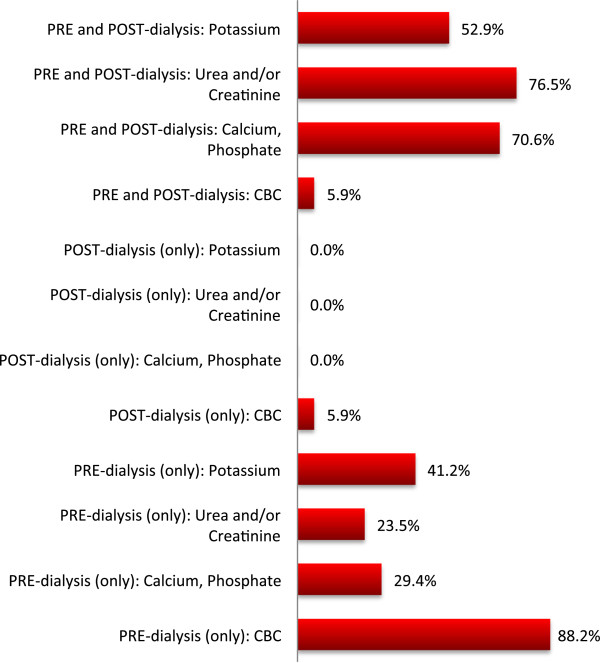
**Timing and nature of routine blood work performed by NHD patients.** Proportion of programs requiring specific blood work pre- and/or post-dialysis.

The majority of programs (82.3%) had weekly or monthly follow-up clinics, of which 58.8% of programs have a dedicated HHD clinic, 35.3% had combined HHD/PD clinic, and 5.9% had a combined modality and pre-dialysis care clinic. All programs reported their clinics are multi-disciplinary in which 88.2% of programs adhered to a standard checklist of items to be addressed each visit. A minority of programs (5.9%) offered telehealth clinics for remote dwelling patients. During the first 3 months of HHD, patients were usually seen monthly (64.7% of programs) and then every 2–4 months thereafter (76.5%). NHD patients were asked to draw routine blood work either once or twice monthly by 35.3% and 58.8% of programs respectively. The frequency of routine blood work for SDHD patients was similar. Patients receiving home conventional HD were doing blood work only once per month in nearly all programs (92.1%). Most programs (70.6%) taught their patients to draw their own blood (including centrifugation), 29.4% of programs had their patients blood work drawn at a local laboratory. Figure 7 summarizes the type and timing of blood work specifically requested for NHD patients, with some tests being performed only pre-dialysis (CBC) and others both pre- and post-dialysis (urea and/or creatinine, and calcium and phosphate). A significant proportion of programs (29.4%) did not monitor post-dialysis calcium and phosphate for their NHD patients.

**Figure 8:**
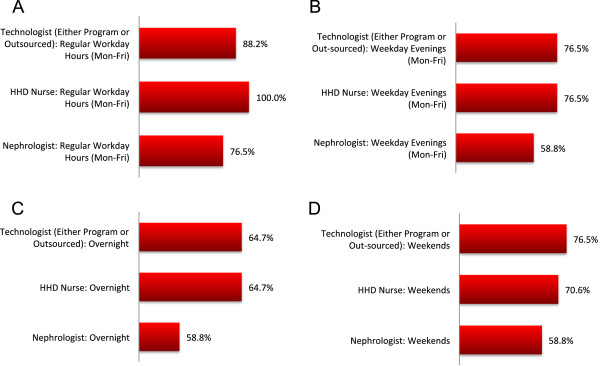
**On-call availability directly to HHD patients. (A)** Monday-Friday during regular working hours, **(B)** Monday-Friday evenings, **(C)** overnight, and **(D)** weekends. The availability of different professionals is not mutually exclusive; a program may provide simultaneous coverage with a nurse *and* technologist, or a nurse *or* a technologist. These data cannot be used to conclude that some programs provide no coverage during certain time periods.

Figure 8 outlines the availability of on-call staff to HHD patients among Canadian HHD programs. The majority of programs had designated nurses, technologists and nephrologists on-call for troubleshooting HHD problems. Whether a nurse and a technologist are on-call was not mutually exclusive so a program may have one or both professionals available at a given time. The questionnaire did not specifically address if no one was on-call during a given time period.

### Medications

Tapering schedules for anti-hypertensive medications and phosphate binders for more frequent HHD patients was predominately managed on an ad-hoc basis (in 76.5% and 64.7% of programs respectively). Fifty-three percent of programs adjusted phosphate binders or add supplemental phosphate to the dialysate based on pre-*and* post-dialysis serum phosphate concentrations, while 47.1% of programs made these decisions based solely on the pre-dialysis phosphate level.

Self-administration of intravenous iron and antibiotics was allowed by 93% and 76.5% of programs. A minority of programs (16.6%) routinely provided their patients using CVCs a first dose of an empiric antibiotic to be kept at home for self-administration at the first sign of infection. Only 37.5% of programs allowed patients to self-treat with plasminogen activator (tPA). The proportion of HHD programs paying for the following medications was: cinacalcet (17.6% of programs pay), supplemental phosphate to dialysate (88.2%), supplemental calcium to dialysate (76.5%), phosphate binders (35.3%), tPA (88.2%), intravenous antibiotics (76.5%), erythropoietin (70.6%), and intravenous iron (88.2%). This questionnaire did not address who does pay for these medications if not the HHD program (ie. hospital pharmacy, patient, private insurer, etc.).

### Non-adherent patients

Almost all programs cited non-adherence issues in their programs. The most frequent was skipped treatments (reported by 82.4% of programs). Other issues included not taking prescribed medications (76.5%), not performing machine maintenance (70.6%), skipping blood work and missing clinics (each by 64.7% of programs), unreachable by telephone for important follow-up and not following direct instructions by on-call personnel (each by 58.8% of programs), and refusing home visits (35.3%). Approximately two-thirds of physician HHD directors agreed with the statement “competent patients are entitled to make decisions that clinicians think are unsafe” even though 70.6% of programs reported having removed a patient from HHD due to non-adherence (against the patient’s wishes). Almost none of the programs have a written policy for dealing with non-adherence (82.4%).

To our knowledge this the first large-scale practice pattern survey for the delivery of HHD across experienced programs with input from multi-disciplinary teams. The fact that considerable practice pattern variation was documented across almost all surveyed domains underscores the paucity of high quality evidence to guide clinical care. These results will serve as a basis of comparison among Canadian HHD programs, as well as benchmark the Canadian experience to international HHD practices. Importantly, these survey results should not be viewed as practice recommendations. The variation described reflects how individual programs have adapted their previous experiences with home-based hemodialysis from an earlier era with their contemporary interest in intensification of hemodialysis (ie. NHD and SDHD) which is more conveniently delivered at home.

This survey explored 11 practice domains yielding considerable data of interest to nephrologists, nurses, technologists, administrators, policy makers and patients. Not all of these results can be discussed in detail so we highlight a few salient observations that warrant broader consideration. First, there was significant variation in uptake of both HHD and PD in Canada. It is notable that success in delivering both HHD and PD can be demonstrated in both academic and community settings with examples of a combined prevalent home therapies population of 35-45% among both types of practices (Table 1). The fact that the prevalence of HHD among 3 of 5 community programs is ~10% also suggests that NHD and SDHD have emerged from the realm of experimental therapies and are well-established mainstream treatments in Canada.

Our results highlight considerable variation in programmatic policy when considering patients such as potential living transplant recipients who might be expected to exit HHD prior to 12 months. This is likely based on a program’s interpretation of data suggesting that the financial breakeven point for managing a patient on HHD occurs after about one year [15]; presumably some programs place a high value on this while others do not. A program’s success with potential living donors actually becoming donors and the time involved in the work-up algorithm may also impact on acceptance to HHD training.

The observation that only a small minority of incident ESRD patients from pre-dialysis clinics, self-care units, and urgent dialysis starters will commence either NHD or SDHD represents a system-wide opportunity to specifically target these populations with interventions aimed to educate and convert these patients to HHD (or home therapies generally).

At the time of this survey, the Canadian standards for HHD water quality had not been published which presumably led to more variation in the action limits for CFU/ml and EU/ml [16]. However, there may be a substantial increase in cost associated with implementing these standards and how this impacts potential HHD patients with surface water as their only feed water source is not known. It is unclear what the quality of life and morbidity/mortality trade-offs are for implementing standards that have not been shown to impact either of those outcomes.

Buttonhole cannulation remains common among Canadian HHD programs and while the AVF is the preferred vascular access, there is a common conviction that it remains preferable to have patients self-manage their dialysis at home irrespective of access. It should be noted that this survey was conducted prior to the publication of the Canadian Society of Nephrology (CSN) clinical practice guidelines on intensive HHD [17, 18]. These guidelines dissuade the preferential use of buttonhole cannulation based on emerging literature suggesting that buttonholes pose an increased risk for local and systemic infectious complications over traditional rope-ladder cannulation. Whether these guidelines will alter practice remains to be seen since buttonhole cannulation is perceived as an important convenience in HHD and the quality of evidence against their use is poor. These guidelines also encourage the use of fistulae over other vascular access, but acknowledge that almost no data specific to intensive HHD supports (or refutes) this stance.

The survey reveals the common practice of prescribing single-needle dialysis among HHD patients, particularly among patients with arteriovenous grafts but also those with fistulae. This is presumably out of concern that frequent cannulation of a graft will eventually wear out the synthetic material resulting in access complications, and the worry that inadvertent needle dislodgement in a double-needle setup could result in rapid exsanguination. Neither fear has born out in almost 2 decades of intensive NHD experience in Canada [19]. While patients performing single-needle HHD may experience the less objective advantages from dialyzing at home (eg. flexible schedule, increased locus of control, quality of life, etc.), the biochemical benefit of this dialysis may be overestimated even for long duration treatments compared with double-needle dialysis. To our knowledge, the trade-offs of single- versus double-needle dialysis in the home intensive HD setting have not been systematically investigated.

While all but 2 of 17 Canadian programs surveyed reimburse some or all of the expenses for plumbing and electrical renovations required to perform HHD, considerable costs for dialysis are still transferred to the patient. These include non-reimbursed renovation expenses, additional utility cost incurred by running dialysis and water treatment equipment, and in many cases, to incidental costs of scales, additional water purifiers, etc. A question of equity versus in-centre dialysis is raised if these expenses are not offset by transportation cost savings, especially since widespread reimbursement for the incremental cost of HHD is uncommon in Canada.

Finally, there is considerable variability in the frequency and timing of routine blood work, especially for NHD patients. This is particularly true for calcium and phosphate which 70% of programs measure pre- and post-dialysis while 30% measure only pre-treatment. This lack of consensus underscores our fundamental lack of understanding how post-dialysis concentrations (especially of phosphate) should be interpreted. The aforementioned CSN guidelines also attest to this knowledge gap by suggesting that dialysate be supplemented with phosphate to keep pre-dialysis phosphate in the normal range and post-dialysis phosphate above 0.40-0.70 mmol/L, although this latter statement is a conditional recommendation based on the theoretical risk of hypophosphatemia and very poor quality evidence [18].

While this survey was systematically designed and had strong national participation in its inception and conduct, there are a number of important limitations to be acknowledged. The survey instrument did not undergo rigorous testing for validity and reliability. Because it was intended to cover broadly many domains, it could not address in detail any one domain without inducing respondent fatigue. As with any survey, reliance on self-reporting by HHD medical directors on behalf of their unit may not reflect the true practice within the program. In an attempt to avoid this bias, program leads were asked to include allied health team members in completing this questionnaire. This survey also does not capture the institutional culture that encourages the uptake of HHD, which is presumably a dominant predictor of HHD success, irrespective of practice pattern. Lastly, the results of this survey are now more than 2 years old and practices may have changed during that time.

## Conclusion

This survey of Canadian practice patterns demonstrates considerable variability in how HHD is conducted. Whether such variation contributes to differential uptake of HHD among programs cannot be concluded from these data. Since programmatic differences in HHD may have an impact on a patient and family’s success within the program, the extent to which programmatic differences in practices contribute to patient dropout and survival in HHD is currently under investigation. In the future, our survey could also be used to explore programmatic differences by patient location (rural versus urban, province, country) and centre type.

## Electronic supplementary material

Additional file 1: **Questionaire.** (PDF 259 KB)
