# Supplementary material for: Programmatic variation in home hemodialysis in Canada: results from a nationwide survey of practice patterns
Source: Can J Kidney Health Dis. 2014 Jun 10;1:11. doi: 10.1186/2054-3581-1-11 (PMC4349311; doi:10.1186/2054-3581-1-11)
Supplement: Supplementary file 1 — Additional file 1: Questionaire. (PDF 259 KB) [file 40697_2014_13_MOESM1_ESM.pdf]

1. Canadian Home Hemodialysis Practice Patterns Survey

Dear Canadian Home Hemodialysis Program Director:

The purpose of this survey is to describe the Home Hemodialysis experience in Canada across several domains.
It intends to capture similarities and variances in our practice patterns by centre. There are no correct and incorrect responses, and specific answers are not intended to reflect any judgement on quality of care.
No individual centres will be publicly singled out. The survey is purposely not anonymous at present so that we can contact you for clarification of your answers as required. All participating physician directors will be listed in any publication. If you are not comfortable being identified, please consider filling out the survey anonymously.

We suggest that responses to many of these questions be answered in collaboration with your nursing and technical team before submitting back to us.

Thank-you again for your participation and we look forward to hearing from you.

2. Contact Information

1. Please fill out the following so we may contact you to clarify individual responses as requ

Name:

Hospital/Program

Address:

City/Town:

Email Address:

Phone Number:

3. Program Information

This section intends to capture the types of home hemodialysis being peformed in your centre

* **1. How many total ESRD patients are cared for by your centre (including all in-centre units, satellite units, peritoneal dialysis and home hemodialysis patients?**

* **2. Of the ESRD dialysis population cared for by your program, how many perform conventional (thrice weekly) in-centre hemodialysis?**

* **3. Of the ESRD dialysis population cared for by your centre, how many currently perform Peritoneal Dialysis?**

* **4. Of the ESRD dialysis population cared for by your centre, how many currently perform in-centre nocturnal hemodialysis?**

5. Of the ESRD dialysis population cared for by your centre, how many currently perform in-centre HD more than 3x/week?

* **6. Of the ESRD dialysis population cared for by your centre, how many currently perform home hemodialysis (HHD)?**

7. In what year did your Home Hemodialysis Program begin?

8. In what year did your Home Hemodialysis Program begin?

* **9. Of the patients in your program performing Home Hemodialysis, how many are doing each of the following:**

Short Daily Hemodialysis (2-3 hours on 5-7 days per week)

Conventional Home Hemodialysis (3-4 hours on 3-4 days per week)

Nocturnal (Nightly) Hemodialysis (6-8 hours on 4-7 nights per week)

Nocturnal (Nightly) Every Other Night Hemodialysis

On a schedule not encompassed by the above options

4. Program Recruitment

This section of the survey has to do with methods of recruiting patients on to your home hemodialysis program

*** 1. Are potential new recruits to the HHD program formally assessed before acceptance by anyone? (check all that apply)**

- ☐ Nephrologist
- ☐ Nurse
- ☐ Technician
- ☐ Dietician
- ☐ Social Worker
- ☐ No formal process

2. Do you accept patients if early program exit is expected (e.g. Living Donor transplant, PD planned, short life expectancy)

- ☐ Yes, regardless of expected time on program
- ☐ No, if time on program expected <6 months
- ☐ No, if time on program expected < 12 months

Other (please specify)

____

*** 3. Do you have a self-care or assisted self-care Hemodialysis unit associated with your program?**

- ☐ Yes
- ☐ No

4. If yes, what percentage of patients choose to leave the self-care/assisted self-care unit to perform home Hemodialysis?

- ☐ <10%
- ☐ 11-25%
- ☐ 26-50%
- ☐ >50%

5. Do you have a specific recruitment strategy for home HD?

- ☐ Yes
- ☐ No (If no skip to next section)

*** 6. Do you have a special education session for patients with advanced CKD about Home HD?**

- [ ] Yes
- [ ] No

Elaborate as necessary

*** 7. Do you have an options educator/specialist/transition coordinator for the patients who "parachute" on to dialysis unknown previously to your program?**

- [ ] Yes
- [ ] No

8. What percentage of your patients with advanced CKD choose to do home HD?

- [ ] <10%
- [ ] 10-20%
- [ ] 20-30%
- [ ] 30-40%
- [ ] >40%

9. What percentage of patients who parachute on to dialysis ultimately perform home HD?

- [ ] <10%
- [ ] 10-20%
- [ ] 20-30%
- [ ] 30-40%
- [ ] >40%

10. Do you use posters in your clinical areas to advertise home HD options?

- [ ] Yes
- [ ] No

11. Does your program use videos to advertise/give information about home HD options?

- [ ] Yes
- [ ] No

12. Do you regularly discuss potential patients for home HD recruitment at HD rounds?

- [ ] Yes
- [ ] No
- [ ] We do not have regular patient care rounds

13. Does your program actively try to encourage patients failing PD to consider a direct transition to Home HD?

- [ ] Yes
- [ ] No

14. Do you have any other unique recruitment strategies for your home HD program? (describe)

5. Program Human Resource Inputs

This section asks questions pertaining to the Human Resource Inputs directly dedicated to your Home HD program. Some of these questions may be best answered in collaboration with the program manager.

1. Which of the following best describes your program in terms of staffing?

- [ ] All allied health resources are strictly dedicated to the HHD program
- [ ] Some allied health members are strictly Home HD program (e.g. nurses) while some are shared with other units like in-centre HD or PD (e.g. pharmacy, techs)
- [ ] Our program shares all allied health resources with other programs with nurses and other team members floating between HHD and programs such as PD

Other (please specify)

[ ]

2. How many Full Time Equivalants (FTE's) of each of the following allied health members are dedicated to your Home HD program at present (please estimate time dedicated to HHD for those team members shared with other programs)?

Nurses	
Dialysis Technologists	
Pharmacists	
Social Workers	
Dieticians	
Clerks/Unit Assistants	
Check here if Program Manager unable to calculate FTE's at present	

3. How many FTE's per additional patient is your program supposed to resource of the following allied health team members (Please estimate time dedicated to HHD program for allied health members that are shared with other programs)?

Nursing	
Dialysis Technologists	
Pharmacists	
Social Workers	
Dieticians	
Clerks/Unit Assistants	
Check here if Program Manager unable to calculate ideal FTE's	

4. Which of the following best describes your Home HD program in terms of nephrologist coverage?

- [ ] 1 nephrologist is responsible for all patients in the HHD program
- [ ] A small grop of nephrologists interested in HHD are responsible for all the patients in the program
- [ ] Nephrologists follow their own patients transitioning on to the HHD program

Other Arrangement for Nephrologist Coverage

[ ]

5. Which of the following best describes your Home HD program in terms of nursing coverage?

- [ ] Primary Nursing Model (Nurses train and subsequently follow their own patients the majority of the time).
- [ ] Shared Nursing Model (Nurses train and follow multiple patients with a collective shared responsibility for all patients).
- [ ] Primary Duties Model (Some nurses always train, some always do follow-up visits and clinics)

Other (please specify)

[ ]

6. Which of the following duties does your dialysis technologist perform? (Check ALL that apply)

- [ ] Assessment of patient for suitability (Technical Component)
- [ ] home assessment
- [ ] Technical teaching of patient during training
- [ ] Set-up of equipment in patient's home
- [ ] Servicing of HD machine
- [ ] Servicing of water treatment equipment
- [ ] On-Call to patients for technical issues (Daily)
- [ ] On-Call to patients for technical issues (24-hour)
- [ ] Water sampling for testing
- [ ] Monitoring of water sample test results

Other (please specify)

[ ]

6. Program Equipment

This section asks questions of the equipment used in your Home HD program. This section is likely best answered in collaboration with your technology team.

1. What type of delivery system (machine) does your program currently have in use? (Check ALL that apply in your program)

- [ ] Fresenius
- [ ] Gambro
- [ ] Bellco (BHC Medical)
- [ ] Mix of different machines (Specify Below)
- [ ] Different machines depending on modality (conventional vs. nocturnal - Specify Below))

Other (please specify)

2. Which of the following best describes your Home NOCTURNAL HD patients who use AV fistulas (check all that apply)?

- [ ] The MAJORITY use single needle technique as per program policy
- [ ] The MINORITY use single needle technique
- [ ] PATIENTS WHO ARE ALONE are required to use single needle technique
- [ ] PATIENTS WITH ACCESS ISSUES use single needle technique
- [ ] A mix of patients use single needle technique depending on prescribing physician

Other (please specify)

3. Which of the following best describes your Home NOCTURNAL HD patients who use AV GRAFTS (check all that apply)?

- [ ] The MAJORITY use single needle technique as per program policy
- [ ] The MINORITY use single needle technique
- [ ] PATIENTS WHO ARE ALONE are required to use single needle technique
- [ ] PATIENTS WITH ACCESS ISSUES use single needle technique
- [ ] A mix of patients use single needle technique depending on prescribing physician
- [ ] We have no AV Graft patients in our program

4. Who is responsible for equipment maintenance and service calls?

- [ ] Primarily Manufacturer (through service contract)
- [ ] Primarily In house (program) technologists
- [ ] Manufacturer for some issues, Program Technologists for others

Other (please specify)

* 5. Do all patients use a Reverse Osmosis system?

- [ ] Yes
- [ ] No
- [ ] Depends on Water Testing

Other (please specify)

* 6. Do all patients use a De-Ionizer (DI)?

- [ ] Yes
- [ ] No
- [ ] Depends on Water Testing

Other (please specify)

* 7. Do all patients use a UV light?

- [ ] Yes
- [ ] No
- [ ] Depends on Water Testing

Other (please specify)

8. Do all patients use a Post Treatment Ultrafilter?

- [ ] Yes
- [ ] No
- [ ] Depends on Water Testing

Other (please specify)

*** 9. Does the program provide/reimburse patients for, a weigh scale?**

- [ ] Yes
- [ ] No

*** 10. Does the program provide a centrifuge?**

- [ ] Yes
- [ ] No
- [ ] Only Out of Town Patients
- [ ] Sometimes (Explain Below)

Why?

11. Does your program re-distribute unopened, unused patient supplies from someone who no longer needs them?

- [ ] Yes
- [ ] No

If yes (please specify)

12. Does your program use "wetness" detectors?

- [ ] Around access cannulation sites
- [ ] On floor beside dialysis machine
- [ ] All of the above
- [ ] None of the above

Other (please specify)

5

6

7. Water for Home Hemodialysis

*** 1. Does the renal program reimburse patients for additional utility expenses for home HD (check all that apply)?**

- [ ] Yes
- [ ] No
- [ ] A fixed monthly stipend is provided (may or may not cover the entire cost)
- [ ] The program provides a letter to patients to write-off their additional utility expenses

If no, how much do you tell patients it will cost?

*** 2. Have patients in your program used well water?**

- [ ] Yes
- [ ] No

If YES: How many litres/minute do you a require a well to be able to deliver? Any other considerations/precautions?

*** 3. Have patients in your program used surface water?**

- [ ] Yes
- [ ] No

Other (please specify)

*** 4. Is there a person in your program designated to regularly test for the following (check ALL that apply):**

- [ ] Microbial Contamination
- [ ] Endotoxin Units
- [ ] Organics/Inorganics
- [ ] None of These

How often is this done (IF Yes to 1 and/or 2)

5. Who is responsible for drawing water samples for the above testing?

- [ ] Patients
- [ ] Dialysis Technologists
- [ ] Equipment Manufacturer (i.e. outsourced)

Other (please specify)

6. What is the maximum visible microbial count that you allow in your product water for home hemodialysis

- [ ] No Specification
- [ ] <50 CFU/mL
- [ ] <100 CFU/mL
- [ ] <150 CFU/mL
- [ ] <200 CFU/mL

Other (please specify)

7. What is the maximum endotoxin concentration that you allow in your product water for home hemodialysis?

- [ ] No specification
- [ ] < 0.25 EU/mL
- [ ] < 0.50 EU/mL
- [ ] < 1.00 EU/mL
- [ ] < 2.00 EU/mL

Other (please specify)

8. Vascular Access

*** 1. What is the preferred choice of access for your HHD patients? (Check ONE)**

- [ ] No "Preferred Access Type"
- [ ] AV Fistula
- [ ] AV Graft
- [ ] Central Venous Catheter

*** 2. Does the ABSENCE of preferred vascular access type preclude initiation of HHD?**

- [ ] Yes
- [ ] No

*** 3. Does the ABSENCE of preferred vascular access DELAY initiation of HHD (e.g. delay training until an AVF matures)**

- [ ] Yes
- [ ] No
- [ ] Sometimes

*** 4. For AVF patients, what percentage are currently useing the BUTTONHOLE cannulation technique (vs. the ROPE LADDER technique)**

- [ ] 0
- [ ] 1-20
- [ ] 21-40
- [ ] 41-60
- [ ] 61-80
- [ ] 81-99
- [ ] 100

*** 5. For AVF patients using BUTTONHOLES, how many sets of BUTTONHOLES are routinely created?**

- [ ] 1 Set (2 Sites)
- [ ] 1.5 sets (3 Sites)
- [ ] 2 Sets (4 Sites)
- [ ] 3 Sets (6 Sites)
- [ ] Buttonholes not routinely used

Other (please specify)

*** 6. Have you had to bring patients back to establish new buttonholes?**

- [ ] Yes
- [ ] No

Other (please specify)

*** 7. Which of the following statements apply to your HHD patients using a Central Venous Catheter? (Check all that apply)**

- [ ] All patients routinely use connecting safety devices (e.g. lockboxes)
- [ ] All patients routinely use TEGO-type (or similar style) connectors to prevent air embolism in the event clamps are not locked
- [ ] None of our patients use any special connectology

Other (please specify)

*** 8. For Central Venous Catheter patients, which solution is ROUTINELY instilled into the CVC lumen between runs?**

- [ ] Saline
- [ ] Heparin
- [ ] Citrate

IF CITRATE or HEPARIN, what concentration?

*** 9. If your program trains >3 days a week, and a patient has a new AV fistula (never used before), do you modify your training regimen to accommodate that?**

- [ ] Yes
- [ ] No
- [ ] Not/Applicable (We never train > 3 days per week)

Other (please specify)

10. What type of needles does your program routinely use for AVF patients that are using rope ladder technique?

- [ ] Teflon/SuperCath/Angiocath
- [ ] Steel
- [ ] Not applicable (no one is using rope ladder technique)

Other (please specify)

11. What type of needles does your program routinely use for AVF patients using buttonhole technique?

- [ ] Teflon/SuperCath/Angiocath
- [ ] Steel
- [ ] Not applicable (No one is using buttonhole technique)

Other (please specify)

12. Does your program require special safety engineered needles for AVF's?

- [ ] Yes
- [ ] No

13. How often are AVF/AVG access flow measurement done for HHD patients?

- [ ] Never
- [ ] More often than once per month
- [ ] Every 2 to 3 months
- [ ] More than every 3 months
- [ ] Depends on the clinical situation

9. Training New Patients

This section asks about routine training practices in your program

*** 1. How many days per week does your program typically train a new patient?**

- ☐ 3
- ☐ 4
- ☐ 5
- ☐ 6

*** 2. How many hours per training day does the patient spend in the training unit on a typical day?**

- ☐ <3
- ☐ 4
- ☐ 5
- ☐ 6
- ☐ 7
- ☐ 8 or more

*** 3. What is the median number of weeks a patient trains for in your program**

- ☐ <4
- ☐ 5-6
- ☐ 7-8
- ☐ 9-10
- ☐ >10

Other (please specify)

4. Which of the following best describes nurse ratios during typical patient training?

- [ ] 1:1 throughout training
- [ ] 2 patients: 1 nurse throughout training
- [ ] 1:1 for some aspects of training, 2:1 for others
- [ ] No typical model defined

Other (please specify)

*** 5. During the first patient run at home, who is present? (check ALL that apply)**

- [ ] Nurse
- [ ] Technologist
- [ ] Both Nurse and Technologist
- [ ] Nobody, Patient typically runs alone
- [ ] Variable, depending on location of patient

*** 6. How many total days are typically allocated for re-training using an AVF or AVG when the patient was initially trained for HHD using a CVC?**

- [ ] 1-3
- [ ] 4-6
- [ ] 7-9
- [ ] >10
- [ ] None (Other arrangements made- list below)

Other (please specify)

7. Which of the following teaching/training tools does your program use? (Check ALL that apply)

- ☐ (Self) Program-generated training manual
- ☐ Industry-Generated training manual (e.g. Fresenius)
- ☐ (Self) Program-generated Teaching Videos
- ☐ Industry-Generated Training Videos

Other (please specify)

5
6

10. Dialysis Prescription

This section asks questions of typical prescriptions of patients in your program

1. For Home HD patients doing CONVENTIONAL (THRICE WEEKLY) HEMODIALYSIS, please indicate the standard initial concentrations of the following constituents:

Na

K

HCO3

Acetate (0 if not used)

Ca

Mg

Glucose

High or Low Flux Dialyzer

What is routine Qb?

What is routine Qd?

2. For patients dialyzing using a Nocturnal (3-6 nights per week) protocol please indicate the initial standard dialysate concentrations of the following constituents.

Na

K

HCO3

Acetate (0 if not used)

Ca

Mg

Glucose

High or Low Flux Dialyzer

What is routine Qb?

What is routine Qd?

3. For patients dialyzing using a SDHD protocol please indicate the initial standard dialysate concentrations of the following constituents

Na

K

HCO3

Acetate (0 if not used)

Ca

Mg

Glucose

High or Low Flux dialyzer

What is routine Qb?

What is routine Qd?

4. Does your program use ultrafiltration profiling on HHD patients?

- [ ] Routinely
- [ ] Occasionally
- [ ] Never

5. Does your program use some sort of Blood Volume Monitoring technique on HHD patients?

- [ ] Yes, on most or all patients (Including nocturnal and extended hours patients)
- [ ] Never
- [ ] Occasionally
- [ ] Conventional HD/SDHD patients only

6. Which of the following does your program routinely do when the desired net ultrafiltration (UF) is zero:

- [ ] Set the machine for a UF of zero.
- [ ] Administer a Normal Saline bolus and set the dialysis machine for an hourly UF rate to remove the bolus volume on that HD session
- [ ] Administer a continuous infusion of saline and set the dialysis machine for a UF rate to continuously remove the infused saline

Other (please specify)

5
6

7. What is the routine heparin anticoagulation for HHD (starting dose, Bolus Units/ Hourly Infusion rate)

11. Home Requirements

This section is comprised of questions regarding home requirements and renovations to start a patient on home hemodialysis in your program. This section may be best answered in collaboration with your technical team.

*** 1. Who is responsible for performing a technical assessment of homes for patients wishing to enter your home hemodialysis program?**

- [ ] Completely Outsourced to 3rd Party Vendors (e.g. Fresenius, Gambro, etc..)
- [ ] Technologist dedicated to your home hemodialysis program
- [ ] Combination of Program Technologist with outsourcing of some tasks (e.g. plumber)
- [ ] Other (please specify)

2. Do nurses routinely perform home assessments in addition to your technical assessments?

- [ ] Yes
- [ ] No

Other (please specify)

3. Who is responsible for performing required RENOVATIONS to patients' homes for patients starting HHD?

- [ ] Completely Outsourced to 3rd Party Vendors
- [ ] Technologist dedicated to your home hemodialysis program
- [ ] Some tasks outsourced (e.g. plumbing), some tasks done by program technologists

Other (please specify)

*** 4. Which of the following best describes the technologist support in your home hemodialysis program?**

- [ ] We have one (or more) technologists that only service the home hemodialysis program
- [ ] We have one or more technologists that support both the home and in-centre programs (i.e. no dedicated support personnel)
- [ ] Technologists are completely outsourced
- [ ] Other (please specify)

*** 5. What is the average per patient cost to your program for home assessment and renovations with each new start?**

- [ ] < $1000
- [ ] $1000 - $1499
- [ ] $ 1500 - $ 1999
- [ ] $2000 - $ 2499
- [ ] > $2500
- [ ] Program does not cover costs

Comment

*** 6. For which of the following would your currently deny accepting a home hemodialysis patient on to your program from a WATER perspective? (Check ALL that apply)**

- [ ] Source of water is a well (i.e. separate from municipal water supply)
- [ ] Source of water is a tank
- [ ] Source of water is surface water
- [ ] Water QUALITY was substandard (i.e. impurities)
- [ ] PLUMBING (e.g. water pressure, piping, drains) was substandard and/or not modifiable
- [ ] Program would not pay for necessary PLUMBING modifications
- [ ] Other (please specify)

*** 7. For which of the following would you currently deny a patient on to your home hemodialysis program for an ELECTRICAL reason? (Check ALL that apply)**

- ☐ No reliable, municipal electrical source (e.g. reliance on generator power)
- ☐ Housing electical system (e.g. wiring, breakers) substandard and/or not modifiable
- ☐ Program would not pay for necessary ELECTRICAL modifications

Other (please specify)

8. Does your program cover costs for additional water treatment equipment? (check ALL that apply)

- ☐ Water Softener
- ☐ Iron Filter
- ☐ UV light
- ☐ No additional water treatment equipment covered

Other (please specify)

9. Does your program pay for home renovations for a patient more than once (ie if they move)?

- ☐ Yes
- ☐ No

10. Does your program provide a service that allows patients to dialyze in RVs/Cottages?

- ☐ Yes
- ☐ No

If yes, who pays for this service?

* 11. Which of the following types of "technologically challenging" patients have you accepted and successfully maintained in your home hemodialysis program? (Check ALL that apply)

- [ ] Rural location (>100 km from your centre)
- [ ] Non-municipal Water Source (Tank or Well)
- [ ] Inadequate Septic System
- [ ] Small Total Living Area
- [ ] No municipal electrical source (e.g. generator power)

Other (please specify)

* 12. Do you require that home patients have an assistant? (Check all that apply)

- [ ] For all patients
- [ ] For selected patients
- [ ] For all doing nightly dialysis
- [ ] Not required at all

13. If you require assistants for some or all of your patients, which best describes the assistants? (Check all that apply)

- [ ] Family or living companions only
- [ ] Paid assistants (paid by program)
- [ ] Paid assistants (paid by 3rd party insurance)
- [ ] Unpaid volunteer assistants
- [ ] Privately paid assistants

Other (please specify)

14. If your program allows for paid assistants, does your program require any special training for that assistant require any additional training besides home HD training?

- [ ] Yes
- [ ] No

If yes, please elaborate

15. Which of the following best describes your program's use of remote monitoring?

- [ ] All patients must use
- [ ] For all overnight dialysis runs
- [ ] For all initially, then selected patients
- [ ] Selected patients only (e.g. without a companion)
- [ ] Never use remote monitoring

Other (please specify)

16. How often are dialysis supplies sent to patients?

- [ ] More frequently than every 2 weeks
- [ ] Every 2 weeks
- [ ] Every 3 to 4 weeks

Other (please specify)

12. Patient Follow-up Schedule

This section of the survey asks questions on how patients are typically followed up after they are performing independent hemodialysis at home

* 1. Which of the following best describes the structure of your home hemodialysis follow-up clinics?

- [ ] Home Hemodialysis Follow-up Clinic? Are we supposed to have those?
- [ ] Physician-Only Clinic
- [ ] Home HD Nurse-Only Clinic
- [ ] Physician and Nurse-Only Clinic
- [ ] Multidisciplinary Allied Health Clinic (MD, Nurse, Dietician, Pharmacy, Social Work, or some combination of these)

Other (please specify)

2. Which of the following best describes your clinic model?

- [ ] Dedicated HHD only clinic
- [ ] Combined HHD/PD clinic
- [ ] Combined HHD/PD/pre-dialysis clinic

Other (please specify)

* 3. How often are TYPICAL patients brought in for clinic in the first 3 months after they begin performing hemodialysis at home?

- [ ] Weekly
- [ ] Every 2-3 Weeks
- [ ] Monthly
- [ ] Every 2 Months
- [ ] Every 3 Months
- [ ] > Every 3 Months
- [ ] Some Combination of Above in Graduated Format
- [ ] Every Patient is Different (no fixed schedule)

Other (please specify)

* 4. How often are TYPICAL, STABLE patients brought in for clinic visits after the first 3 months of hemodialysis at home?

- [ ] Monthly
- [ ] Every 2-4 Months
- [ ] Every 5-9 months
- [ ] Every 10-12 months
- [ ] Annually
- [ ] Every Patient is Different (No Fixed Schedule)

Other (please specify)

* 5. How often does your program hold a home hemodialysis clinic?

- [ ] Weekly
- [ ] Every 2 Weeks
- [ ] Monthly

Other (please specify)

6. Do you offer telehealth clinics for remote HHD patients?

☐ Yes

☐ No

☐ Not applicable

7. How often do nurses perform home visits for established patients on the HHD program?

☐ Never

☐ Only for first 1-2 runs at home

☐ For first 1-2 runs, then subsequently at a fixed schedule

☐ For first 1-2 runs, then on an as-needed, ad hoc basis

Other (please specify)

[ ]

8. Which of the following personnel are available to return calls DIRECTLY to HHD patients at the stated hours for patients BETWEEN clinic visits? (Check ALL that apply for each row)

	Regular Workday Hours (Mon-Fri)	Weekday Evenings (Mon-Fri)	Overnight	Weekends
Nephrologist	☐	☐	☐	☐
HHD Nurse	☐	☐	☐	☐
Technologist (Either Program or Out-sourced)	☐	☐	☐	☐

9. How often are patients brought back into the HHD unit for a pre-scheduled "re-training" or "technique audit".

☐ Never

☐ Once in the first year, then never again

☐ Yearly

☐ Only if there is an event/occurrence

☐ Only at HHD nurse/nephrologist request

Other (please specify)

[ ]

10. Which best describes your NOCTURNAL patients in terms of obtaining their bloodwork?

	CBC	Calcium, Phosphate	Urea and/or Creatinine	Potassium
PRE-dialysis (only)	☐	☐	☐	☐
POST-dialysis (only)	☐	☐	☐	☐
PRE and POST-dialysis	☐	☐	☐	☐

11. How do HHD patients get their bloodwork drawn?

☐ Most patients draw their own, including spinning their own blood at home with a centrifuge when required

☐ Most patients go into their local lab to get bloodwork drawn

☐ Drawn at the time of clinic visit

Other (please specify)

12. Which best describes your HHD program in terms of routine bloodwork SCHEDULE (not including special circumstances like acute illness, losing a set-up, etc...)?

	WEEKLY when starting, then MONTHLY	BIWEEKLY	MONTHLY	QUARTERLY	ONLY BEFORE CLINICS	ONLY AS NECESSARY
NOCTURNAL HHD patients	☐	☐	☐	☐	☐	☐
CONVENTIONAL HHD patients	☐	☐	☐	☐	☐	☐
SHORT DAILY HHD patients	☐	☐	☐	☐	☐	☐

Other (please specify)

13. When patients are assessed in the HHD clinic, is there a standard checklist of items to be addressed every visit?

☐ Yes

☐ No

13. Medications

This section primarily deals with how patients administer medications at home

1. How do your HHD patients typically administer the following medications (Check all that apply)?

	Patients never self-administer this medication at home	Intravenous Push	Intravenous Pump	Subcutaneous
Iron	☐	☐	☐	☐
EPO	☐	☐	☐	☐
Antibiotics	☐	☐	☐	☐
tPa	☐	☐	☐	☐

Other comments

2. What medications are paid for by the HHD program (check all that apply)?

- ☐ Intravenous iron
- ☐ Epo
- ☐ Intravenous antibiotics
- ☐ tPA
- ☐ Phosphate binders
- ☐ Calcium spike
- ☐ Phosphate spike
- ☐ Cinacalcet

Other (please specify)

3. Do patients in your HHD program with central venous catheters have a 1st dose of antibiotics at home to be given with the first sign of infection?

- ☐ Yes
- ☐ No

4. Which best describes how your program handles anti-hypertensive medications once more frequent HHD is started?

- [ ] Weaning of medications by protocol
- [ ] Weaning of certain medications by protocol (e.g. continue beta blockers or ACE/ARB's in some patients)
- [ ] Patients given weaning parameters on a case by case basis (ad hoc)

Other (please specify)

5. After how many weeks of initially performing frequent HHD is the patient's blood pressure evaluated to determine of medication dose adjustment is necessary

- [ ] Weekly until stable BP on minimal medication
- [ ] Every 2 Weeks
- [ ] Every 4 Weeks
- [ ] Every 8 Weeks
- [ ] On case by case basis
- [ ] Only at Clinic Visits

Other (please specify)

6. How does your program deal with phosphate binders once frequent dialysis is started?

- [ ] Weaning by fixed protocol on weekly basis (i.e. check Ca/PO4 weekly and wean according to lab values)
- [ ] Weaning by protocol on monthly basis (i.e. check Ca/PO4 monthly and wean according to lab values)
- [ ] Weaning schedule on ad hoc basis

Other (please specify)

7. After how many weeks of initially performing HHD is the patient's phosphate evaluated to determine if phosphate binder dose adjustment is necessary?

- ☐ 1
- ☐ 2
- ☐ 3
- ☐ 4
- ☐ 4-8
- ☐ >8

Other (please specify)

8. Which best describes how your program deals with phosphate balance for frequent HHD patients?

- ☐ Adjust binders/additives based solely on pre-HD phosphate
- ☐ Adjust binders/additives based on a combination of pre and post-HD phosphate

Other (please specify)

14. Non-Adherent Patients

This section pertains to how your program deals with the "non-adherent" patient.

1. Has non-adherence been an issue in your HHD program in the following domains? (check ALL that apply)

- ☐ Not performing HD/RO machine maintenance
- ☐ Not following direct instructions of on-call personnel
- ☐ Missing Clinic Visits
- ☐ Not Taking Prescribed Medications
- ☐ Skipping Bloodwork
- ☐ Unreachable by Telephone
- ☐ Skipping Treatments
- ☐ Refusing home visits

Other (please specify)

2. Does your HHD program have a written policy for how to deal with the non adherent patient?

- ☐ No
- ☐ Yes

If YES, Describe

3. Do you believe that patients deemed competent to perform HHD and make medical decisions are allowed to make "bad" choices?

- ☐ Yes, If a patient is competent, they are entitled to make decisions which clinicians may deem "unsafe"
- ☐ No, These patients are the clinician's ultimate responsibility and likely should be removed from the HHD program

4. Have you ever removed a patient (against their wishes) from your program for non-adherence?

- ☐ Yes
- ☐ No

5. Which of the following patients would you consider removing from your HHD program (against their wishes)?

- ☐ Misses 25% of their standard bloodwork
- ☐ Misses 50% of their standard bloodwork
- ☐ Misses 75% of their standard bloodwork
- ☐ Never does bloodwork (>12 months)
- ☐ We would not remove a patient for this issue

6. Which of the following patient would you consider removing from your program?

- ☐ Never attends follow-up visits
- ☐ Misses 50% of follow-up visits
- ☐ Misses 75% of follow-up visits
- ☐ We would not remove a patient for this issue

7. Which of the following patients would you consider removing from your program?

- ☐ Misses 25% of machine/RO maintenance
- ☐ Misses 50% of machine/RO maintenance
- ☐ Misses 75% of machine/RO maintenance
- ☐ Misses all of machine/RO maintenance
- ☐ We would not remove a patient for this issue

8. Which of the following patients would you consider removing from your program re: Water Sampling/Testing?

- ☐ Misses 25% of routine water sampling/testing
- ☐ Misses 50% of routine water sampling/testing
- ☐ Misses 75% of routine water sampling/testing
- ☐ Misses all of routine water sampling/testing
- ☐ Our program performs this function on behalf of patients
- ☐ We would not remove a patient from the program for this issue

9. Would you consider removing a patient from your program if they consistently didn't maintain any HD records from their home treatments?

- [ ] Yes
- [ ] No

10. If you ever have let a patient (or patients) from your program, what were the primary reasons?

11. Are service logs (i.e. records of patient tuns) linked to HHD program funding?

- [ ] Yes
- [ ] No

15. Anything we missed?

If there are any domains of HHD practice that we have missed you would like to comment on, please do so here.

1. Comments
